# Serum Biomarkers of Cardiovascular Remodelling Reflect Extra-Valvular Cardiac Damage in Patients with Severe Aortic Stenosis

**DOI:** 10.3390/ijms21114174

**Published:** 2020-06-11

**Authors:** Laura Bäz, Gudrun Dannberg, Katja Grün, Julian Westphal, Sven Möbius-Winkler, Christian Jung, Alexander Pfeil, P. Christian Schulze, Marcus Franz

**Affiliations:** 1 Department of Internal Medicine I, University Hospital Jena, Friedrich Schiller University, 07747 Jena, Germany; Laura.Baez@med.uni-jena.de (L.B.); Gudrun.Dannberg@med.uni-jena.de (G.D.); Katja.Gruen@med.uni-jena.de (K.G.); Julian.Westphal@med.uni-jena.de (J.W.); Sven.Moebius-Winkler@med.uni-jena.de (S.M.-W.); Christian.Schulze@med.uni-jena.de (P.C.S.); 2 Division of Cardiology, Pulmonology and Vascular Medicine, Medical Faculty, University Duesseldorf, 40225 Duesseldorf, Germany; Christian.Jung@med.uni-duesseldorf.de; 3 Department of Internal Medicine III, University Hospital Jena, Friedrich Schiller University, 07747 Jena, Germany; Alexander.Pfeil@med.uni-jena.de

**Keywords:** aortic stenosis, staging, extra-valvular cardiac damage, cardiovascular remodelling, biomarker, risk stratification

## Abstract

In patients with aortic stenosis (AS), a novel staging classification of extra-valvular left and right heart damage with prognostic relevance was introduced in 2017. The aim of the study was to evaluate the biomarkers of cardiovascular tissue remodelling in relation to this novel staging classification. Patients were categorized according to the novel staging classification into stages 0 to 4. The levels of matrix metalloproteinase 9 (MMP-9), tissue inhibitor of metalloproteinases 1 (TIMP-1), B and C domain containing tenascin-C (B^+^ Tn-C, C^+^ Tn-C), the ED-A and ED-B domain containing fibronectin (ED-A^+^ Fn, ED-B^+^ Fn), endothelin 1 (ET-1) and neutrophil gelatinase-associated lipocalin (NGAL) were determined in serum by ELISA. There were significantly decreased serum levels of MMP-9 and increased levels of B^+^ Tn-C and C^+^ Tn-C when comparing stages 0 and 1 with stage 2, with no further dynamics in stages 3 and 4. In contrast, for TIMP-1, C^+^ Tn-C, ED-A^+^ Fn, ET-1 and NGAL, significantly increased serum levels could be detected in stages 3 and 4 compared to both stages 0 and 1 and stage 2. ED-A^+^ Fn and ET-1 could be identified as independent predictors of the presence of stage 3 and/or 4. To the best of our knowledge, this is the first study identifying novel serum biomarkers differentially reflecting the patterns of left and right heart extra-valvular damage in patients suffering from AS. Our findings might indicate a more precise initial diagnosis and risk stratification.

## 1. Introduction

Degenerative aortic valve stenosis (AS), the most frequently occurring acquired valvular heart disease, shows increasing prevalence rates in the aging society [1,2]. Within the last decade, the disease has gained more attention, since both diagnosis and therapy have tremendously changed. With respect to diagnosis, echocardiography remains the gold standard [3], but the classical definition of severe AS, which was designated as high-gradient aortic stenosis (HGAS), has been extended. Thus, two further entities, namely low-gradient aortic stenosis (LGAS) and paradoxical low-flow, low-gradient aortic stenosis (PLFLGAS), have been introduced, referring to the importance of stroke volume for correct severity grading [1,4,5,6,7]. The latter is mandatory to avoid underestimation of the stenosis, resulting in treatment delay, and also with respect to the fact that the different entities are of prognostic relevance [8].

Current guidelines recommend trans-catheter aortic valve implantation (TAVI) in high- and moderate-risk patients after heart team discussion, while recent large randomized trials clearly showed the non-inferiority or even superiority of TAVI also in low-risk elderly patients [9,10]. As a consequence, patients eligible for TAVI will represent a more and more heterogeneous collective in terms of, among other factors, age, comorbidities or special anatomical states.

To date, the most important question in the management of AS patients was the choice of treatment mode (surgery vs. TAVI). In contrast, future challenges will be, first, the definition of certain subgroups of patients which benefit from treatment mostly realized by TAVI; and second, the choice of the timepoint of treatment within the disease continuum of each individual patient [11]. To meet these challenges, there is a need to get deeper insights into the extent of both myocardial and pulmonary vascular damage, which determine patient symptoms, clinical outcome and prognosis at different timepoints, in addition to the initially causative AS itself.

Recently, a novel staging classification of severe aortic stenosis has been introduced.

By dividing the patients’ collective of the Partner 2 trials into four stages, taking the individual presence of left ventricular, left atrial, pulmonary vascular and right ventricular involvement into account [12], the authors could clearly show that rising stages assessed before the treatment of AS are associated with an increased long-term mortality.

On the molecular basis, all these processes of extra-valvular damage can be subsumed by the term cardiovascular tissue remodelling [13]. Besides the alterations in cardiac myocytes themselves, there is an activation and trans-differentiation of cardiac fibroblasts to cardiac myofibroblasts [14], or an activation of endothelial cells with consecutive vascular remodelling, mainly driven by endothelin-1 (ET-1) [15] and inflammatory pathways [16]. In that context, among others, neutrophil gelatinase-associated lipocalin (NGAL) has been identified as an important mediator of cardiovascular inflammation [17]. All the sub-processes of cardiovascular remodelling are accompanied by a structural and functional reorganisation of the cardiac extracellular matrix (cECM) [18], starting with the degradation of pre-existing extracellular structures, mainly realized by Matrix metalloproteinases (MMP) and their endogenous inhibitors, the so-called tissue inhibitors of metalloproteinases (TIMP) [19]. Among these, MMP-9 and TIMP-1 play certain roles in the cardiovascular system [20]. Interestingly, NGAL, as an important mediator of inflammation, could be shown to regulate cECM reorganisation by directly inhibiting MMP-9 activity [17]. In the next step following cECM degradation, there is a de novo synthesis of matrix components, e.g., collagens, entailing the re-occurrence of fetal variants in the cell-adhesion-modulating proteins fibronectin (Fn) and tenascin-C (Tn-C). For the latter, a functionally relevant disease-associated re-expression could be demonstrated for a variety of cardiovascular disorders, both in terms of a stable tissue deposition as well as liberation into the circulation, and might therefore serve as excellent biomarkers or even therapeutic targets [21,22,23,24,25].

### Aim of the Study

Thus, the purpose of the current study was to determine the serum levels of certain preselected surrogate markers of cardiovascular remodelling and their subprocesses in a real-world moderate-to-high-risk collective of AS patients undergoing TAVI. The latter were grouped according to the novel staging classification of extra-valvular cardiac damage published in 2017 [12] for dedicated analysis of biomarker levels between the different staging groups.

## 2. Results

### 2.1. Characterization of the Study Population

**Figure 1:**
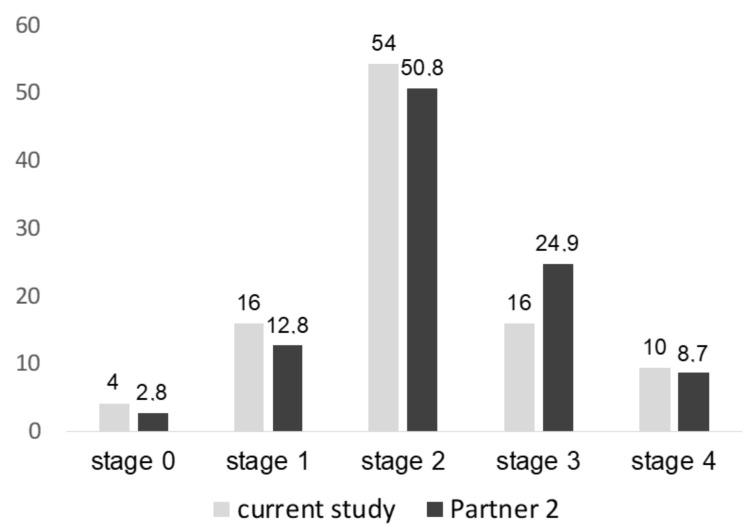
Distribution of the different stages of extra-valvular damage in this study compared to the results in the Partner 2 trial. Except for stage 3, for all stages, the percentage of patients per stage was similar between the studies.

In this prospective single-centre study, 94 moderate-to-high-risk elderly patients suffering from AS and undergoing a TAVI procedure (mean age 78.3 ± 7.3 years, 46.8% male, mean Society of Thoracic Surgeons (STS)-Score 4.4 ± 2.8%) were compared to 37 cardiovascular risk patients without manifest cardiovascular disorder. Baseline characteristics including relevant comorbidities of patients and controls are given in Table 1. In all AS patients, a transfemoral approach was chosen for TAVI and carried out successfully. In 47.9% of the patients, a balloon-expandable, and, in 52.1%, a self-expendable, valve was implanted. According to the purpose of our study, the patients were divided into different groups according to the 2017 staging classification [12]. Figure 1 shows the distribution of the different stages, each compared to the results of the Partner 2 trials (Figure 1). Referring to the small number of subjects, especially in stages 0 and 4, we defined the following subgroups: group 1 = stage 0 + 1 (*n* = 19); group 2 = stage 2 (*n* = 51); group 3 = stage 3 + 4 (*n* = 24). The baseline characteristics in comparison of these groups are given in Table 2.

### 2.2. Comparative Analysis of Clinical Characteristics between the Different Staging Groups and Disease Entities

Detailed results including *p*-values for all clinical parameters, compared between the different staging groups, are given in Table 2.

**Figure 2:**
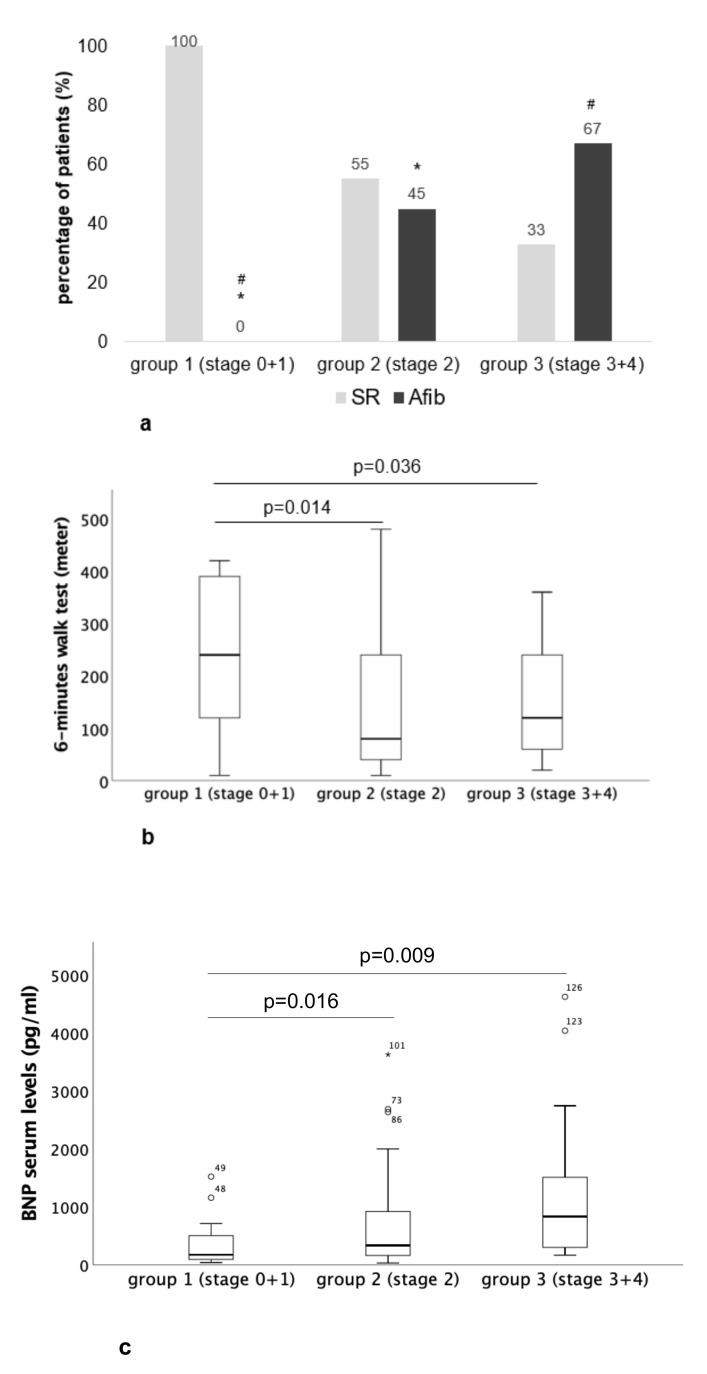
Clinical characteristics assessed in this study in comparison between the three different staging subgroups: (**a**) heart rhythm: sinus rhythm versus atrial fibrillation (*,^#^ indicate significant differences between the groups with a *p* value <0.05), (**b**) 6-minutes’ walk test and (**c**) BNP serum levels.

When comparing all clinical characteristics assessed in this study, significant differences between group 1 (stage 0 + 1) and group 2 (stage 2) could only be evidenced for STS score (higher values in group 2, *p* = 0.016), heart rhythm (sinus rhythm versus atrial fibrillation, higher frequency of atrial fibrillation in group 2, *p* < 0.001, Figure 2a), 6-minutes’ walk test (6MWT) (lower distances in group 2, *p* = 0.014, Figure 2b), Brain natriuretic peptide (BNP) serum levels (higher values in group 2, *p* = 0.016, Figure 2c) and ≥ II° mitral regurgitation (higher frequency in group 2, *p* = 0.006).

In group 3 (stage 3 + 4) versus group 1, there were significant differences for STS score (higher values in group 3, *p* = 0.001), heart rhythm (sinus rhythm versus atrial fibrillation, higher frequency of atrial fibrillation in group 3, *p* < 0.001, Figure 2a), 6MWT (lower distances in group 3, *p* = 0.036, Figure 2b), BNP serum levels (higher values in group 3, *p* = 0.009, Figure 2c), Glomerular filtration rate (GFR) ≤ 30 mL/min (higher frequency in group 3, *p* = 0.048), ≥ II° mitral regurgitation (higher frequency in group 3, *p* = 0.003), ≥ II° tricuspid regurgitation (higher frequency in group 3, *p* < 0.001), Right ventricular (RV) dysfunction (higher frequency in group 3, *p* = 0.003), Systolic pulmonary artery pressure (sPAP) (higher values in group 3, *p* = 0.023) as well as the frequency of HGAS (higher in group 1, *p* = 0.005) and LGAS (lower in group 1, *p* = 0.014).

When comparing group 3 versus group 2, the only significant differences could be evidenced for GFR ≤ 30 mL/min (higher frequency in group 3, *p* = 0.034), interventricular septal thickness at end diastole (IVSd) ≥ 12 mm (higher frequency in group 2, *p* = 0.035), ≥ II° tricuspid regurgitation (*p* < 0.001), RV dysfunction (*p* < 0.001), as well as the frequency of HGAS (higher in group 2, *p* < 0.001) and LGAS (lower in group 2, *p* = 0.010).

With respect to the three different entities of AS (HGAS, LGAS, PLFLGAS), the only significant differences could be shown between the HGAS and the LGAS group for 6MWT (larger distances in the HGAS group, *p* = 0.037), right ventricular dysfunction (lower frequency in the HGAS group, *p* < 0.001) and staging group (higher stages in the LGAS group, *p* = 0.001).

### 2.3. Analysis of Biomarkers of Cardiovascular Remodelling in Comparison Between Controls and the Different Staging Groups

The serum levels of the eight preselected surrogate biomarkers of cardiovascular remodeling, as described above, were compared first between the control group (*n* = 37) and the entire TAVI patients’ collective (*n* = 94), and second, between the three predefined staging subgroups representing the extent of extra-valvular cardiac damage in the AS patients. When comparing controls and TAVI patients, there were significantly increased serum levels for TIMP-1, B^+^ Tn-C, ED-B^+^ Fn, NGAL and ET-1 and decreased serum levels for MMP-9 (*p* < 0.05 for all biomarkers). Detailed results including *p* values are given in Table 3.

**Figure 3:**
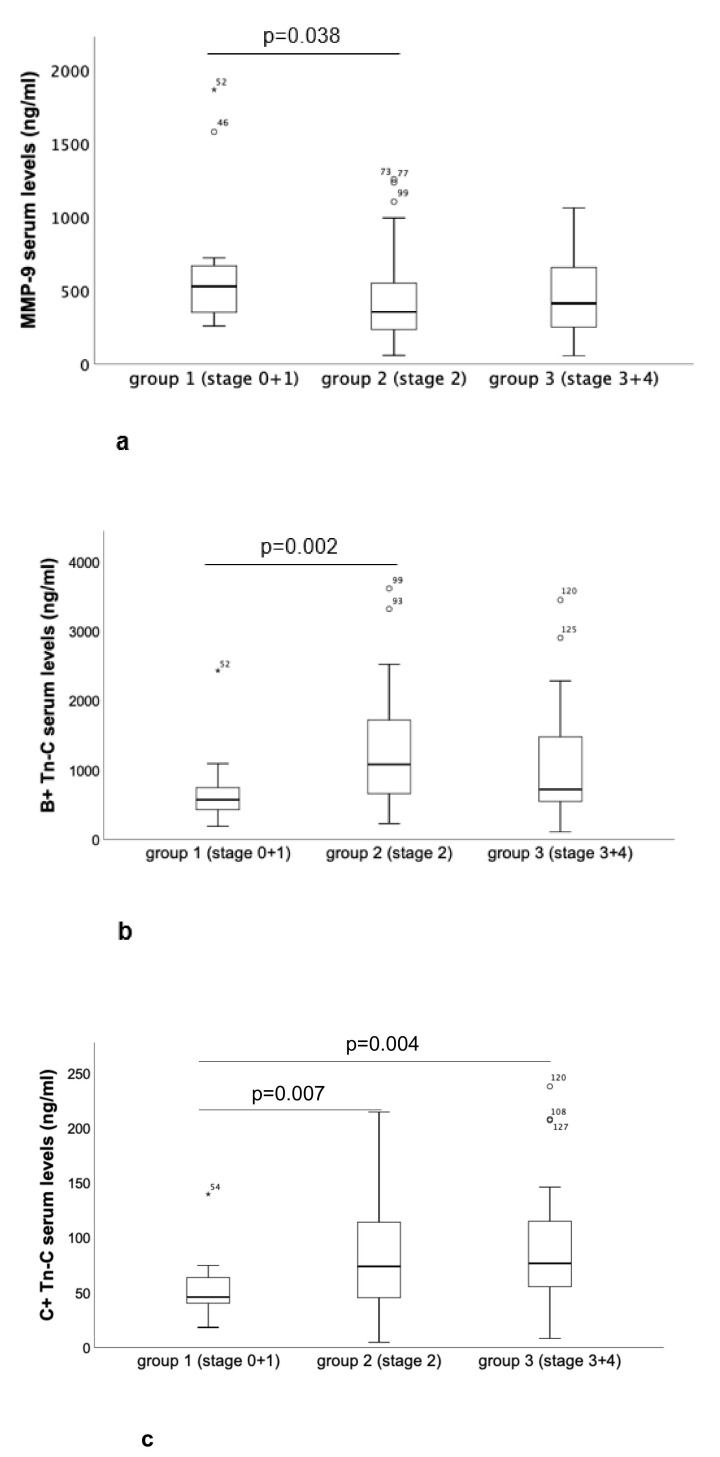

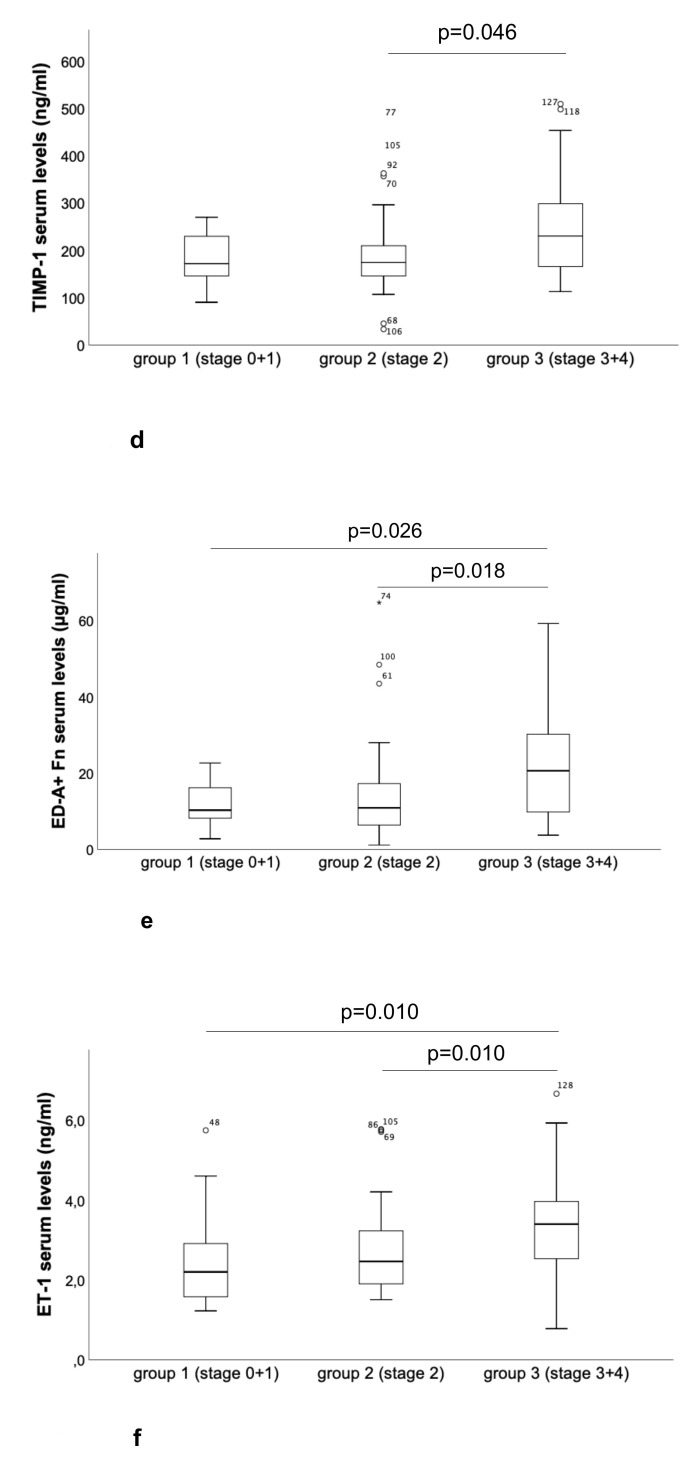

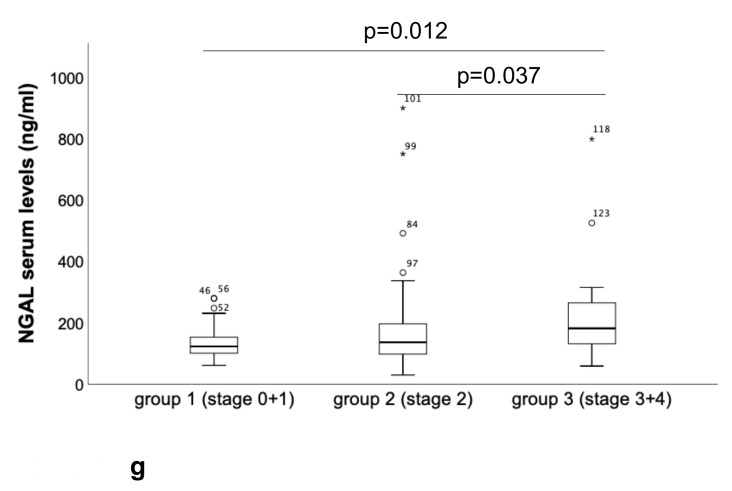
Differences in serum biomarkers in comparison with the three different staging subgroups within the TAVI collective: (**a**) MMP-9, (**b**) B^+^ Tn-C, (**c**) C^+^ Tn-C, (**d**) TIMP-1, (**e**) ED-A^+^ Fn, (**f**) ET-1 and (**g**) NGAL. The given *p* values indicate statistical significance (Mann–Whitney-U test, *p* value < 0.05).

Comparison of the three subgroups within the TAVI collective revealed a significant decrease in the serum levels of MMP-9 and a significant increase in B^+^ Tn-C and C^+^ Tn-C between group 1 (stage 0 + 1) and group 2 (stage 2), with no further dynamics between group 2 and group 3 (stage 3 + 4) (Figure 3a–c). For TIMP-1, ED-A^+^ Fn, ET-1 and NGAL serum levels, no significant differences occurred between group 1 and 2. In contrast, there were significantly increased levels for these biomarkers in comparison with both group 3 and 2 as well as group 3 and 1 (Figure 3d–g). Detailed results including *p* values are given in Table 4.

Besides staging groups, also the three different entities of AS (HGAS, LGAS, PLFLGAS) were compared with respect to the serum levels of all eight biomarkers measured in this study. In summary, no significant differences could be observed for all biomarkers (*p* = n.s.; data not shown). With respect to differences between males and females in the TAVI patients’ collective, the only biomarker showing significant differences is MMP-9 (males: 336.5 ± 253.4 ng/mL versus females: 426.6 ± 360.4 ng/mL; *p* = 0.014; median ± SD). For all other parameters, there were no significant differences (*p* = n.s.).

### 2.4. Parameters Predicting an Advanced Stage of Extravalvular Cardiac Damage in the Study Population

To test the predictive value of TIMP-1, ED-A^+^ Fn, ET-1 and NGAL for the probability of an advanced stage (stage 3 or 4), logistic regression analysis (backward elimination WALD) including eight relevant covariates was performed as described below. Only ED-A^+^ Fn (OR: 1.134; CI: 1.044–1.231; *p* = 0.003) and ET-1 (OR: 1.674; CI: 1.029–2.723; *p* = 0.038) could be identified as independent predictors.

## 3. Discussion

The idea and motivation for the current study was to divide a real-world, moderate-to-high-risk patients’ collective undergoing TAVI into different groups according to the 2017 staging classification and to analyse the serum levels of certain biomarkers of cardiovascular remodelling in these groups and between the disease entities (phenotypes) in comparison to a control group [1,12]. Of note, the control group described above in detail is younger compared to the TAVI patients’ collective. This fact has to be considered as a limitation of the current study and should be taken into account when interpreting the results.

Most of the preselected biomarkers measured by us showed significant alterations in serum levels when comparing the entire patients collective with the control group. This finding speaks well for their functional involvement in cardiovascular tissue remodelling, associated with AS development. However, since not all of these markers are cardiac-specific, due to the multimorbidity of the elderly AS patients, an extra-cardiac origin cannot be completely excluded [26,27]. Most strikingly, in the AS patients we found decreased levels of MMP-9 and increased levels of TIMP-1, reflecting a steady state of remodelling in which there is a pro-fibrotic cECM accumulation more prominent than degradation and turnover [19]. The certain role of these two isoforms of the MMP/TIMP system in cardiovascular disorders has been extensively described in the past [20,28,29].

Interestingly, when comparing males and females within the TAVI patients’ collective with respect to serum biomarker levels, only MMP-9 revealed significantly elevated levels in females compared to males. This is in contrast to the findings of other groups not reporting on relevant gender differences in MMP-9 serum levels [30,31]. Nevertheless, to our best knowledge, this particular aspect has not been investigated in detail in patients with severe AS.

In addition, we found significantly increased serum levels for special fetal-splicing variants of Tn-C and Fn. These variants are overexpressed in crucial steps of embryonic cardiovascular development, are virtually absent in healthy adult organs, and are abundantly re-expressed in a wide range of cardiovascular diseases [20,21,22,23,24,28,32,33]. NGAL, classically known as a biomarker of renal failure, could be evidenced as a useful circulating reflector of cardiovascular inflammation, including atherosclerosis development [17,34]. In accordance, we could show significantly elevated levels in AS patients compared to controls. This is of special interest since NGAL has been described to inhibit MMP-9 function, and thereby cECM degradation, which goes in line with the significant reduction in MMP-9 levels in this study [17].

The last important biomarker observed to be significantly elevated is ET-1. The molecule mediates vasoconstriction and is crucially involved in vascular remodelling occurring in a variety of cardiovascular disorders, in particular pulmonary hypertension [35,36] or cardiac allograft vasculopathy following heart transplantation [37]. Taking the first part together with this, the majority of biomarkers analysed in our study qualify as circulating reflectors of cardiovascular remodelling in general, and also in moderate-to-high-risk elderly AS patients eligible for TAVI.

The most interesting question to be answered was the differential association of biomarker levels to the different disease entities (phenotypes) of AS and especially the different stages of extra-valvular cardiac damage. Surprisingly, we did not observe any differences for all biomarkers when comparing HGAS, LGAS and PLFLGAS. This is in contrast to our initial hypothesis, in which we expected significant differences for several biomarkers, at least in the LGAS group, when compared to HGAS or PLFLGAS. Thus, in contrast to our findings, Fabiani and colleagues could describe differences between different entities of aortic stenosis, at least with respect to pro-fibrotic microRNAs [38]. Similar studies focussing on surrogate biomarkers of cECM remodelling were not available from the literature until now.

In contrast to the entities, when comparing the different staging groups [12], we observed impressive alterations in biomarker serum levels linked to cardiovascular remodelling in terms of extra-valvular damage progression. Concerning the differences in clinical parameters between the different staging groups, the majority of the observed findings given in Table 2 were not surprising, since they largely contribute to the definition of the stage itself, e.g., atrial fibrillation or relevant mitral regurgitation for stage 2. With respect to the biomarker analyses, most strikingly, we could describe the following two clusters. First, there were significant alterations in MMP-9 (decrease), B^+^ and C^+^ Tenascin-C (increase) when comparing staging group 2 (stage 2) with staging group 1 (stage 0 or stage 1). Interestingly, when comparing staging group 3 (stage 3 or stage 4) with staging group 2, there were no further dynamics for the three biomarkers. These results reflect a serum liberation of the mentioned biomarkers at the particular timepoint of disease progression when extra-valvular damage switches from left ventricular to left atrial involvement. In accordance, alterations in the serum levels of MMP-9 and Tenascin-C variants reflecting myocardial tissue remodelling have been recently described in hypertensive heart disease [23,29]. Nevertheless, the association explicitly with left atrial remodelling is novel and should also serve as an impetus for further studies on the cellular level.

The second notable cluster of biomarkers consisted of TIMP-1, ED-A^+^ Fn, ET-1 and NGAL, for which we could detect significantly increased serum concentrations, when comparing staging group 3 (stage 3 + 4) with lower staging groups. This finding speaks for a distinct role of these biomarkers for the transition from stage 2 to stage 3 or 4, which means the development of pulmonary hypertension on the basis of pulmonary vascular remodelling and consecutive right heart dysfunction or failure due to right ventricular pressure overload. These findings are in line with recent studies on a certain role of these biomarkers, especially for pulmonary vascular remodelling [15,36,39]. As a result of binary logistic regression analysis, only ED-A^+^ Fn and ET-1 could be identified as independent predictors of staging group 3 (stage 3 + 4), representing advanced extra-valvular cardiac damage. Therefore, these particular biomarkers can be suggested as novel parameters contributing to individual risk stratification and prognosis prediction in patients suffering from AS. The crucial role of ET-1 for vascular remodelling, mainly in the context of pulmonary hypertension, has been extensively described in the literature [15,36,37]. The particular role of ED-A^+^ Fn has been identified recently by our group for vascular remodelling processes linked to cardiac allograft vasculopathy development or pulmonary hypertension [23,39,40,41].

In conclusion, the current study could show that certain key molecules of cardiovascular remodelling qualify as circulating markers of disease progression in elderly moderate-to-high-risk AS patients eligible for TAVI.

Nevertheless, there is a certain aspect that should be discussed as a possible limitation of the current study: some comorbidities, e.g., diabetes or COPD, were significantly different between the groups of AS patients and controls, with higher prevalence in the AS patients’ group. Therefore, the serum levels of the biomarkers assessed by us might be influenced by comorbidities. For instance, in patients with diabetes, relevant alterations in circulating Tenascin-C, MMP-9, ET-1 or NGAL could be observed [42,43,44]. In COPD patients, some of the biomarkers investigated by us, e.g., Tenascin-C, fibronectin, ET-1 or NGAL, have been recently shown to be of potential diagnostic or prognostic impact [45,46,47,48]. Naturally, these aspects should be taken into account when interpreting the results of the current study.

Besides the panel of biomarkers related to cardiovascular ECM remodelling and inflammation investigated in our study, there is, of course, a variety of supplemental biomarkers possibly contributing to a more precise diagnosis of AS severity or even outcome prediction. Among others, asymmetric dimethylarginine (ADMA), homocysteine, plasminogen activator or lipoproteins, have been shown to serve as promising candidates in that context [49,50,51,52,53].

## 4. Materials and Methods

### 4.1. Study Population

This prospective single-centre study included 94 patients with severe AS (all patients revealed tricuspid morphology of the aortic valve) and 37 patients at increased cardiovascular risk but without clinically significant cardiovascular disorder. The patients were admitted to the Department of Internal Medicine I of the University Hospital Jena and included in the clinical registry designated as Jenaer Aortenklappenregister (acronym: JAKR). All patients and controls gave written informed consent for participation before inclusion. The study was approved by the local ethics committee of the Medical Faculty of the Friedrich Schiller University Jena (registration number: 4815-06/16). The investigation was conducted in accordance to the principles of the current version of the Declaration of Helsinki and good clinical practice guidelines. All patients underwent detailed clinical, laboratory, functional and imaging analysis according to local standard operating procedures. The control group consisted of patients at increased cardiovascular risk in which coronary artery disease was excluded by invasive coronary angiography and transthoracic echocardiography, not revealing relevant structural or valvular heart disease or signs of heart failure. Baseline characteristics of the patients and the control group are shown in Table 1.

### 4.2. Stages of Extra-Valvular Cardiac Damage According to the 2017 Staging Classification

In 2017, Généreux and co-workers implemented a new staging classification describing the extent of extra-valvular cardiac damage in AS patients: stage 0 = no cardiac damage; stage 1 = left ventricular damage (increased LV mass index >115 g/m^2^ for males and >95 g/m^2^ for females; E/e’ >14; LV ejection fraction <50%); stage 2 = left atrial or mitral damage (indexed left atrial volume >34 mL/m^2^; moderate to severe mitral regurgitation; atrial fibrillation); stage 3 = pulmonary vasculature or tricuspid damage (systolic pulmonary hypertension ≥ 60 mmHg; moderate to severe tricuspid regurgitation); stage 4 = right ventricular damage (moderate to severe right ventricular dysfunction) [12].

### 4.3. Blood Samples

Blood samples were collected from all participants. Collection tubes were centrifuged within 20 min and serum was transferred into special low binding tubes (Protein LoBind, Eppendorf AG, Hamburg, Germany) and stored at -80°C after snap freezing in liquid nitrogen, to reduce artificial protein degradation. To fulfil quality standards, repeated freeze-thaw-cycles were strictly avoided.

### 4.4. Routine Laboratory Parameters and Quantification of Serum Biomarker Levels

Laboratory parameters measured for clinical routine were determined according to local standard operating procedures. Serum levels of matrix metalloproteinase 9 (MMP-9), tissue inhibitor of metalloproteinase 1 (TIMP-1), B domain containing tenascin-C (B^+^ Tn-C), C domain containing tenascin-C (C^+^ Tn-C), endothelin 1 (ET-1) and neutrophil gelatinase-associated lipocalin (NGAL) were determined using the following commercially available ELISA assays, which are well validated and established in our research laboratory: Human MMP-9 Immunoassay, R&D Systems GmbH, Wiesbaden, Germany; Human TIMP-1 Immunoassay, R&D Systems GmbH, Wiesbaden, Germany; Tenascin-B Large (FNIII-B) ELISA, IBL International GmbH, Hamburg, Germany; Tenascin-C Large (FNIII-C) ELISA, IBL International GmbH, Hamburg, Germany; Endothelin-1 Immunoassay, R&D Systems GmbH, Wiesbaden, Germany; Human Lipocalin-2/NGAL Immunoassay, R&D Systems, Wiesbaden, Germany. All protocols were performed according to the instructions of the manufacturer. For quantification of ED-A^+^ Fn and ED-B^+^ Fn serum levels, commercial ELISA assays are not available. Therefore, we used the protocol recently developed and validated in our group (capturing: gelatine; detection: antibodies IST-9 (ED-A^+^ Fn) or C-6 (ED-B^+^ Fn)) [22].

### 4.5. Statistics

Statistical analyses were performed by using IBM SPSS statistical software, version 25.0 (IBM SPSS Statistics for Windows. Armonk, NY, USA). Data are expressed as mean/median ± standard deviation as appropriate. Mann–Whitney-U test was used to test for significant differences in clinical characteristics as well as biomarker levels between the different groups.

To identify independent predictors for the probability of an advanced stage of extra-valvular damage (stage 3 or 4), a multivariate regression analysis was performed by using a binary logistic model (backward elimination method: Wald). The presence of the stages 3 or 4 was defined as the dependent variable. After group comparison using the Mann–Whitney-U test between group 1 and 3 or group 2 and 3 respectively, we defined the parameters showing *p* < 0.1 (*n* = 8) as covariates: brain natriuretic peptide (BNP) serum levels, six-minutes’ walk test (6MWT) results in meters, B^+^ Tn-C, MMP-9, ED-A^+^ Fn, TIMP-1, ET-1 and NGAL. Thereafter, multistep backward elimination (removal threshold *p* > 0.10) of independent variables was performed. A *p*-value ≤ 0.05 was defined as statistically significant.

## 5. Conclusions

In the current study, we could identify two biomarker clusters differentially reflecting left and right heart involvement in the disease continuum of valvular cardiomyopathy in patients with severe aortic stenosis and consecutive heart failure, as assessed by the staging of extra-valvular cardiac damage. Whereas B^+^/C^+^ Tenascin-C and MMP-9 alterations reflect the switch of extra-valvular cardiac damage from left ventricular to left atrial involvement, TIMP-1, ED-A^+^ Fn, ET-1 and NGAL reflect the further transition from left heart involvement to pulmonary hypertension and consecutive right heart dysfunction. In particular, elevated levels of ED-A^+^ Fn and ET-1 independently predict an advanced stage of extra-valvular damage and can be suggested as novel biomarkers for individual risk stratification before TAVI. Our findings might indicate a more precise initial diagnosis and risk stratification and could be an impetus for further clinical studies including larger patient numbers as well as basic research to elucidate the functional role of the identified key molecules of AS associated cardiovascular remodelling.

## Figures and Tables

**Table 1:** Baseline characteristics of the patients (*n* = 94) and the control group (*n* = 37) and corresponding *p* values (Mann–Whitney-U test). The control group consisted of patients at increased cardiovascular risk but without structural heart disease or heart failure; coronary artery disease has been excluded by invasive coronary angiography. The TAVI (trans-catheter aortic valve implantation) collective represents a typical cohort of elderly patients suffering from severe symptomatic aortic stenosis exhibiting a moderate to high surgical risk.

Parameter	TAVI Collective (*n* = 94)	Controls (*n* = 37)	*p*-value
**Age (mean ± SD**, **years)**	78.3 ± 7.3	66.2 ± 6.5	<0.001
**Male (%)**	46.8	35.1	0.227
**Society of Thoracic Surgeons (STS)-Score (mean ± SD)**	4.4 ± 2.8	N/A	
**New York Heart Association (NYHA) I (%)**	7.4	N/A	
**NYHA II (%)**	29.8	N/A	
**NYHA III (%)**	58.5	N/A	
**NYHA IV (%)**	4.3	N/A	
**NYHA ≤ II (%)**	37.2	N/A	
**NYHA > II (%)**	62.8	N/A	
**Angina pectoris (%)**	31.9	71.4 (*n* = 35)	<0.001
**Coronary Artery Disease (CAD) (%)**	65.9	0	<0.001
**Peripheral Artery Disease (PAD) (%)**	13.8	N/A	
**Diabetes (%)**	44.7	18.9	0.008
**Chronic Obstructive Pulmonary Disease (COPD) (%)**	24.5	2.7	0.004
**Atrial fibrillation (%)**	41.5	18.9	0.015
**6-minutes’ walk test (6MWT) (mean ± SD**, **meters)**	168 ± 133	N/A	
**Glomerular filtration rate (GFR) ≤ 30 mL/min (%)**	13.8	0	0.018
**Dialysis (%)**	5.3	0	0.154
**Brain natriuretic peptide (BNP) ≥ 100 pg/mL (%)**	91	5.6 (*n* = 36)	<0.001
**Left ventricular ejection fraction (LVEF) (%)**	55.7 ± 13.8	67.8 ± 6.9	<0.001
**Interventricular septal thickness at end-diastole (IVSd) ≥ 12 mm (%)**	92 (*n* = 87)	52.2 (*n* = 23)	<0.001
**Mitral regurgitation ≥ II° (%)**	26.6	2.7	0.002
**Tricuspid regurgitation ≥ II° (%)**	18.1	2.7	0.022
**Right ventricular (RV) Dysfunction (%)**	9.6	0	0.052
**Systolic pulmonary artery pressure (sPAP) ≥ 35 mmHg (%)**	77.6 (*n* = 58)	0 (*n* = 36)	<0.001
**Stage 0–4 (%):**			
**0**	4.3	N/A	
**1**	16	N/A	
**2**	54.3	N/A	
**3**	16	N/A	
**4**	9.6	N/A	
**Aortic stenosis (AS) entity (%)**			
**High-gradient aortic stenosis (HGAS)**	72.3	N/A	
**Low gradient aortic stenosis (LGAS)**	17	N/A	
**Paradoxical low flow LGAS (PLFLGAS)**	10.6	N/A	
**Type of TAVI Prosthesis**			
**Edwards SAPIEN 3**, **ballon-expandable (%)**	47.9	N/A	
**CoreValve^TM^Evolut^TM^ R**, **self-expandable (%)**	44.7	N/A	
**ACURATE neo^TM^**, **self-expandable (%)**	7.4	N/A	

**Table 2:** Baseline characteristics of the patients (*n* = 94) in comparison between the three staging groups: group 1 (stage 0 + 1; *n* = 19), group 2 (stage 2; *n* = 51) and group 3 (stage 3 + 4; *n* = 24). The corresponding *p* values (Mann–Whitney-U test) between the different groups reveals that the majority of parameters did not show significant differences (boldness indicates significant *p* values ≤0.05).

Parameter	Staging Group 1 (*n* = 19)	Staging Group 2 (*n* = 51)	Staging Group 3 (*n* = 24)	*p*-value Group 1 vs. 2	*p*-value Group 1 vs. 3	*p*-value Group 2 vs. 3
**Age (mean ± SD**, **years)**	76.5 ± 6.9	79 ± 7.7	78.2 ± 6.9	0.202	0.641	0.422
**Male (%)**	42.2	49	45.8	0.609	0.809	0.798
**STS score (mean ± SD)**	2.9 ± 1.7	4.4 ± 2.5	5.4 ± 2.7	0.016	0.001	0.084
**NYHA I (%)**	0	9.8	8.3	0.160	0.203	0.839
**NYHA II (%)**	31.6	33.3	20.8	0.890	0.428	0.271
**NYHA III (%)**	63.2	52.9	66.7	0.447	0.813	0.265
**NYHA IV (%)**	5.3	3.9	4.2	0.807	0.867	0.960
**NYHA ≤ II (%)**	31.6	43.1	29.2	0.383	0.866	0.250
**NYHA > II (%)**	68.4	56.9	70.8	0.383	0.866	0.250
**Angina pectoris (%)**	42.1	35.3	16.7	0.603	0.068	0.101
**CAD (%)**	57.9	62.7	79.2	0.713	0.136	0.158
**PAD (%)**	10.5	15.7	12.5	0.586	0.843	0.718
**Diabetes (%)**	31.6	54.9	33.3	0.106	0.904	0.105
**COPD (%)**	31.6	27.5	12.5	0.736	0.131	0.152
**Atrial fibrillation (%)**	0	45.1	66.7	**<0.001**	**<0.001**	0.083
**6MWT (mean ± SD**, **meters)**	260 ± 140	141 ± 132	107 ± 81	**0.014**	**0.036**	0.558
**GFR ≤ 30 mL/min (%)**	5.3	9.8	29.2	0.549	**0.048**	**0.034**
**Dialysis (%)**	0	3.9	12.5	0.385	0.114	0.168
**BNP ≥ 100 pg/mL (%)**	68.4 (*n* = 18)	93.9 (*n* = 49)	100 (*n* = 22)	**0.016**	**0.009**	0.239
**LVEF (%)**	60.4 ± 11.3	56.7 ± 12.1	49.7 ± 17.1	0.272	0.057	0.130
**IVSd** **≥ 12 mm (%)**	94.4 (*n* = 18)	95.9 (*n* = 49)	80 (*n* = 20)	0.797	0.194	**0.035**
**Mitral regurgitation ≥ II° (%)**	0	31.4	37.5	**0.006**	**0.003**	0.602
**Tricuspid regurgitation ≥ II° (%)**	0	0	70.8	1.0	**<0.001**	**<0.001**
**RV Dysfunction (%)**	0	0	37.5	1.0	**0.003**	**<0.001**
**sPAP** **≥ 35 mmHg (%)**	50 (*n* = 6)	73.3 (*n* = 30)	90.9 (*n* = 22)	0.264	**0.023**	0.116
**AS entity (%)**						
**HGAS**	84.2	82.4	41.7	0.856	**0.005**	**<0.001**
**LGA)**	5.3	11.8	37.5	0.423	**0.014**	**0.010**
**PLFLGAS**	10.5	5.9	20.8	0.505	0.369	0.052
**Edwards SAPIEN 3**, **ballon-expandable (%)**	47.4	51	41.7	0.790	0.712	0.454
**CoreValve^TM^Evolut^TM^ R**, **self-expandable (%)**	47.4	41.2	50	0.644	0.865	0.476
**ACURATE neo^TM^**, **self-expandable (%)**	5.3	7.8	8.3	0.711	0.698	0.942

**Table 3:** Serum levels of the eight preselected biomarkers investigated in this study in comparison between the control group (*n* = 37) and the entire AS patients’ collective (*n* = 94). There were significant differences (Mann–Whitney-U test, *p* < 0.05) for MMP-9, TIMP-1, B^+^ Tn-C, ED-B^+^ Fn, ET-1 and NGAL (boldness indicates significant p values ≤0.05).

Biomarker	Controls (*n* = 37)	AS-Patients (*n* = 94)	*p*-value
**MMP-9 (ng/mL**, **median ± SD)**	602.05 ± 305.17	399.95 ± 323.6	**0.003**
**TIMP-1 (ng/mL**, **median ± SD)**	159.1 ± 42.66	177.75 ± 88.68	**0.018**
**B^+^ Tn-C (ng/mL**, **median ± SD)**	362.11 ± 380.05	794.05 ± 775.53	**<0.001**
**C^+^ Tn-C (ng/mL**, **median ± SD)**	66.08 ± 27.37	70.03 ± 51.96	0.366
**ED-A^+^ Fn (μg/mL**, **median ± SD)**	8.82 ± 10.22	13.31 ± 12.08	0.116
**ED-B^+^ Fn (μg/mL**, **median ± SD)**	3.06 ± 2.10	5.25 ± 3.23	**0.012**
**ET-1 (ng/mL**, **median ± SD)**	1.35 ± 0.38	2.49 ± 1.19	**<0.001**
**NGAL (ng/mL**, **median ± SD)**	83.14 ± 32.98	142.64 ± 146.42	**<0.001**

**Table 4:** Serum levels of the eight preselected biomarkers investigated in this study in comparison with the three staging groups of AS patients (*n* = 94): group 1 (stage 0 + 1; *n* = 19), group 2 (stage 2; *n* = 51) and group 3 (stage 3 + 4; *n* = 24). The corresponding *p* values (Mann–Whitney-U test) between the different groups reveal certain differences between the groups (boldness indicates significant *p* values ≤0.05).

Biomarker	Staging Group 1 (*n* = 19)	Staging Group 2 (*n* = 51)	Staging Group 3 (*n* = 24)	*p*-value Group 1 vs. 2	*p*-value Group 1 vs. 3	*p*-value Group 2 vs. 3
**MMP-9 (ng/mL**, **median ± SD)**	528.11 ± 418.53	353.60 ± 288.08	411.94 ± 292.48	**0.038**	0.271	0.413
**TIMP-1 (ng/mL**, **median ± SD)**	172.19 ± 53.99	174.32 ± 80.74	230.44 ± 112.44	0.853	0.056	**0.046**
**B^+^ Tn-C (ng/mL**, **median ± SD)**	571.19 ± 483.34	1081.68 ± 788.11	720.93 ± 841.37	**0.002**	0.065	0.343
**C^+^ Tn-C (ng/mL**, **median ± SD)**	45.88 ± 26.12	73.77 ± 52.69	76.53 ± 59.31	**0.007**	**0.004**	0.729
**ED-A^+^ Fn (μg/mL**, **median ± SD)**	10.30 ± 5.95	10.91 ± 11.90	20.65 ± 14.19	0.838	**0.026**	**0.018**
**ED-B^+^ Fn (μg/mL**, **median ± SD)**	5.05 ± 2.73	5.16 ± 3.50	6.22 ± 3.07	0.890	0.353	0.367
**ET-1 (ng/mL**, **median ± SD)**	2.20 ± 1.24	2.47 ± 1.06	3.40 ± 1.28	0.146	**0.010**	**0.010**
**NGAL (ng/mL**, **median ± SD)**	122.84 ± 67.24	136.41 ± 159.12	181.57 ± 158.85	0.561	**0.012**	**0.037**

## Abbreviations

6MWT	6 minutes’ walk test
Afib	atrial fibrillation
B^+^ Tn-C	B domain containing tenascin-C
BNP	brain natriuretic peptide
C+ Tn-C	C domain containing tenascin-C
CAD	coronary artery disease
COPD	chronic obstructive pulmonary disease
ECM ED-A^+^ Fn	extracellular matrix ED-A domain containing fibronectin
ET-1	endothelin 1
GFR	glomerular filtration rate
HGAS	high gradient aortic stenosis
IVSd	interventricular septum thickness in diastole
LGAS	low gradient aortic stenosis
LVEF	left ventricular ejection fraction
MMP-9	matrix metalloproteinase 9
NGAL	neutrophil gelatinase associated lipocalin
NYHA	New York Heart Association
PAD	peripheral artery disease
PLFLGAS	paradoxical low flow low gradient aortic stenosis
RV	right ventricle
SD	standard deviation
sPAP	systolic pulmonary artery pressure
SR	sinus rhythm
STS	Society of Thoracic Surgeons
TAVI	transcatheter aortic valve implantation
TIMP-1	tissue inhibitor of metalloproteinase 1
